# Understanding the role of age and U.S. acculturation factors on the relationship between allostatic load and cancer mortality risk in Hispanic Americans

**DOI:** 10.1007/s10552-026-02162-z

**Published:** 2026-04-22

**Authors:** Jessica Amezcua, Valeria Miranda, Melissa Lopez-Pentecost, Jessica Y. Islam, Marlo M. Vernon, Kathleen O’Connor, Justin Xavier Moore

**Affiliations:** 1 Markey Cancer Center, University of Kentucky, Lexington, KY USA; 2 University of Kentucky College of Medicine, Lexington, KY USA; 3 Sylvester Comprehension Cancer Center, University of Miami, Miami, FL USA; 4 Cancer Epidemiology Program, H. Lee Moffitt Cancer Center and Research Institute, Tampa, FL USA; 5 Georgia Cancer Center, Medical College of Georgia, Augusta University, Augusta, GA USA; 6 Center for Health, Engagement, & Transformation, Department of Behavioral Science, University of Kentucky, Lexington, KY USA

**Keywords:** Allostatic Load, Hispanic, Cancer Mortality

## Abstract

**Purpose:**

Despite growing recognition of stress-related health inequities, the role of nativity and acculturation in shaping the relationship between chronic stress and cancer outcomes among Hispanic populations remains poorly understood. The purpose of this study was to examine whether nativity factors (United States (US) citizenship status and length of time residing in the US modify the association between allostatic load (AL), a measure of chronic physiologic stress, and cancer mortality among Hispanic adults.

**Methods:**

We performed a prospective cohort analysis using data from the National Health and Nutrition Examination Survey (NHANES) 1999 – 2010, linked to National Death Index with follow-up through December 31, 2019. Survey-weighted Cox proportional hazards models were used to estimate hazard ratios (HR) and 95% confidence intervals (CIs) for cancer mortality, including interaction and age-stratified analyses by US citizenship status, length of time in the US, and country of birth. Fully adjusted models included age, sex, education, year interviewed, smoking status, history of heart attack, and congestive heart failure.

**Results:**

Among 7,299 Hispanic adults, 2,835 (33.4% weighted) had high AL. Among Hispanic adults aged ≥ 60 years, individuals with US citizenship and low AL (HR = 3.13; 95% CI = 1.37–7.12), as well as those with US citizenship and high AL (HR = 3.32; 95% CI = 1.18–9.35), experienced more than a three-fold increased risk of cancer mortality compared with non-US citizens with low AL. When examining AL by length of time residing in the US among Hispanic adults aged ≥ 60 years, those with low AL and more than 10 years in the US (HR = 6.32; 95% CI = 1.33–29.90) and those with high AL and more than 10 years in the US (HR = 6.34; 95% CI = 1.17–34.43) had approximately a six-fold increased risk of cancer mortality compared with adults with low AL and less than 10 years in the US.

**Conclusion:**

Cancer mortality risk among Hispanic adults appears to be driven primarily by older age and nativity-related factors rather than AL risk. These findings highlight the need for future research to more fully examine how structural, social, and immigration-related contexts intersect with aging to influence cancer outcomes among Hispanic populations.

**Supplementary Information:**

The online version contains supplementary material available at 10.1007/s10552-026-02162-z.

## Introduction

### Cancer disparities in Hispanic communities

In 2019, one-third of all Hispanic/Latina/o/x (henceforth, Hispanic) people living in the United States (US), were born outside of the country [1]. According to the 2020 US Census, Hispanic individuals are the second-largest ethnic group in the US, representing 19.1 percent of the total population, this percentage is projected to grow to 26.9% by 2060 [2]. Despite being the youngest demographic in the US, with a median age of 30 years, cancer has emerged as the leading cause of death in this group, with 176,600 new cases and 46,500 deaths reported in 2021 [3]. As this community continues to grow and age, the potential cancer burden becomes a pressing concern [4]. While current data indicate that the Hispanic population experience lower overall cancer incidence rates compared to non-Hispanic White people, they are more often diagnosed at later stages, resulting in lower survival rates [5].

### Acculturation, assimilation, and the role of stress

Historically, acculturation models in the US have been perceived as one-dimensional, with the belief that Anglo cultural traits were superior to those of immigrant groups [6]. Early American anthropologists introduced concepts such as “amicable acculturation,” where more “civilized” and “enlightened” cultures engaged in cooperative and friendly cultural exchanges, and “piratical acculturation,” which referred to hostile and forced cultural exchanges, particularly in early societies deemed “savage” or “barbaric” [7].

Contemporary Hispanic populations illustrate the complexities of acculturation and navigating dual cultural identities within American society [8]. Positive health outcomes are often associated with successful integration, especially when adequate resources and support systems are accessible. However some individuals face pressures that can lead to negative health behaviors, such as substance use and poor dietary choices, as well as adverse health outcomes, including pregnancy complications [9]. Negative health behaviors and outcomes are often exacerbated by various acculturation-related stressors, including family separation, low socioeconomic status, language barriers, racism, and discrimination [10]. Additionally, many Hispanic individuals begin their careers in sectors such as farming, construction, and food service, often starting in lower-wage jobs due to limited education and English proficiency [11]. These jobs frequently involve hazardous conditions, such as extreme heat exposure, poor hydration, and cultural barriers that limit access to necessary resources and protections, further increasing the risk of negative health outcomes [12]. Despite these challenges, greater access to educational opportunities and resources offers potential for upward socioeconomic mobility and improved access to supports for healthy behaviors [13]. Social integration varies significantly based on generation, immigration status, and nationality, influencing factors such as English proficiency and educational achievement. The acculturation process can result in cultural conflict and involves psychological processes, including social learning, stress, coping, identity, resilience, mental illness, and conflict [14].

### Allostatic load and risk of cancer death

AL refers to the body’s wear and tear resulting from the prolonged inability to deactivate stress mediators, ultimately compromising immunity and contributing to disease [15]. AL is measured using biomarkers reflecting various regulatory systems relevant to disease risks including cardiovascular, immune, metabolic, and neuroendocrine systems [15]. Studies have linked negative health behaviors like substance abuse, inadequate sleep, and obesity with increased AL risk, especially among individuals coping with chronic stress, depression, or anxiety [16].

Environmental and social factors, including poor living conditions, overcrowded housing, pollution, and limited access to green spaces, further exacerbate chronic stress, disrupting the hypothalamic–pituitary–adrenal (HPA) axis and increasing cortisol levels [17]. This can contribute to greater body mass index (BMI), and persistent inflammation, all of which are associated with elevated cancer risk [18].

Discrimination and racism experienced by Hispanic populations also can intensify stress, contributing to higher AL and exacerbate negative behavioral changes that heighten cancer risk [19]. Addressing social and environmental factors that contribute to higher AL is crucial. Understanding the biological impacts of stress and acculturation will enable healthcare providers to develop strategies to reduce cancer risks and improve health outcomes for Hispanic populations. Limited research has explored the correlation between nativity and acculturation indicators and AL; and to our knowledge, no studies have specifically examined the role of US citizenship status and length of time in the US on AL and cancer among Hispanic adults living in the US. This study seeks to explore the moderating roles of US citizenship status and length of time in the US on the relationship between AL and cancer mortality in a representative sample of Hispanic people.

## Methods

We conducted a prospective cohort study using data from National Health and Nutrition Examination Survey (NHANES) with mortality follow-up through linkage to the National Death Index. The NHANES is a cross-sectional survey administered by the Centers for Disease Control and Prevention’s (CDC) National Center for Health Statistics (NCHS). The NHANES study sample represents non-institutionalized U.S residents, including about 5,000 participants per year. To ensure a representative sample, NHANES oversamples individuals aged 60 and older, as well as non-Hispanic Black, and Hispanic people. The survey offers a comprehensive approach by combining interviews that collect data on demographics, lifestyle, and health-related factors with standardized physical examinations conducted in specially equipped Mobile Exam Centers (MECs).

We linked NHANES data with follow-up data on mortality (through December 31, 2019) using the National Death Index (NDI) and NHANES linked files curated by NCHS. We examined the association between AL and cancer mortality among Hispanic participants. To evaluate the effects of US citizenship status and length of time in the US, we limited our analysis to participants surveyed between 1999 and 2010, as NHANES surveys before 1999 did not collect data on citizenship status or length of time in the US. In 1999, NHANES transitioned to a continuous annual survey, collecting data in two-year cycles.

Participants were excluded sequentially based on prespecified criteria. Specifically, 46,080 participants were excluded because they were pregnant at the time of examination or younger than 18 years of age, 11,360 participants were excluded due to missing AL biomarkers or incomplete mortality follow-up, and 15,201 participants were excluded because they participated in NHANES cycles prior to 1999, when information on U.S. citizenship status and length of time in the US was not collected. After applying these exclusion criteria, the final analytic sample consisted of 7,229 Hispanic participants. The sample selection process and corresponding exclusions are summarized in the study flow diagram.

Given the relatively low incidence of cancer mortality among younger adults, we conducted age-stratified analyses among participants aged 40–59 years and ≥ 60 years to better capture cancer-relevant risk and assess whether associations differed by age group. Participants younger than 40 years were included in the full analytic sample but were not included in the age-stratified tables, as long-term cancer mortality outcomes are less interpretable in this group.

### Exposure: allostatic load

AL is measured using a combination of biomarkers and physiological indicators that reflect the impact of chronic stress on various body systems [20]. Although there is no gold standard for measurement, common systems assessed include cardiovascular, metabolic and immune [21]. We defined high AL as having more than three abnormal measures among the following nine biomarkers: Body Mass Index (BMI), C-reactive protein (CRP), systolic blood pressure (SBP), diastolic blood pressure (DBP), glycated hemoglobin, total cholesterol, serum triglycerides, serum creatinine, and serum albumin [22] (Supplemental Table 1). Sex-specific distributions were established for each of these allostatic biomarkers to identify high-risk thresholds among the entire NHANES study sample from 1999 – 2010 (including all racial and ethnic groups). High risk-cutoffs were defined as values above the sex-specific 75th percentile for BMI (31.24 kg/m^2^ in females; 30.33 kg/m^2^ in males), CRP (0.49 (mg/dL) in females; 0.30 mg/dL in males), SBP(131.41 mmHg in females; 132.15 mmHg in males), DBP (77.70 mmHg in females; 81.07 mmHg in males), glycohemoglobin (5.55% in females; 5.56% in males), total cholesterol (227.70 mg/dL in females; 225.11 mg/dL in males), serum triglycerides (154.97 mg/dL in females; 192.02 in males), and serum creatinine (79.60 mol/L in females; 103.21 mol/L in males). Serum albumin was classified as high risk when values were below the 25th percentile (4.35 g/dL in females; 4.35 g/dL in males). Each biomarker was then assigned a score: 1 for high risk and 0 for low risk. The overall AL score was calculated from summing the individual biomarker scores, resulting in a total score ranging from 0 to 9. Participants with a total AL score of 3 or higher were classified as having a high AL.

**Table 1:** Socio-demographic characteristics and personal health by allostatic load status, among 7,299 participants using National Health Examination Survey (NHANES) 1999–2010 and follow up through December 31, 2019

	Total Sample N = 7,299	High AL N = 2,835	Low AL N = 4,464	p value
	*Presented as N (%) or Mean (SD)*			
AL Total Score, Mean (SD)*	1.94 (0.04)	4.0 (0.03)	0.91 (0.02)	< 0.0001
**Female Sex**	3644 (48.0)	1490 (49.9)	2154 (47.1)	0.0647
**Age, Mean (SD)**	39.2 (0.3)	47.3 (0.5)	35.2 (0.3)	< 0.0001
*Age Category*				< 0.0001
< 40 years	3289 (56.8)	576 (33.2)	2713 (68.7)	
40–59 years	2163 (31.9)	1038 (45.0)	1125 (25.3)	
60 + years	1847 (11.3)	1221 (21.9)	626 (6.0)	
*Time Period (Year)*				< 0.0001
1999–2002	2399 (32.7)	800 (27.1)	1599 (35.5)	
2003–2006	1857 (28.1)	717 (29.7)	1140 (27.3)	
2007–2010	3043 (39.2)	1318 (43.2)	1725 (37.2)	
*Citizen Status*				< 0.0001
US Citizen	4373 (58.2)	1886 (65.0)	2487 (54.7)	
Non-US Citizen	2892 (41.4)	940 (34.7)	1952 (44.8)	
*Length of Time in US*				< 0.0001
< 10 Years	1405 (21.3)	304 (12.7)	1101 (26.2)	
10 +	2785 (37.9)	1296 (45.0)	1489 (34.3)	
Missing	3109 (40.4)	1235 (42.2)	1874 (39.4)	
*Country of Birth*				0.1832
United States	2921 (37.7)	1164 (39.3)	1757 (36.9)	
Mexico	3144 (35.7)	1141 (33.5)	2003 (36.8)	
Other	1228 (26.5)	526 (27.1)	702 (26.2)	
*Mother Country of Birth*				0.0061
United States	906 (12.4)	370 (5.1)	536 (7.3)	
Mexico	1958 (26.8)	636 (8.7)	1322 (18.1)	
Other	394 (34.5)	132 (1.8)	262 (3.6)	
Missing	4041 (55.3)	6161 (84.4)	5179 (71.0)	
*Father Country of Birth*				0.0007
United States	796 (10.9)	331 (4.5)	465 (6.4)	
Mexico	2041 (28.0)	671 (9.2)	1370 (18.8)	
Other	407 (5.6)	132 (1.8)	275 (9.82)	
Missing	4055 (55.6)	6,165 (84.5)	2,110 (28.9)	
*Education*				< 0.0001
< High school	3953 (46.3)	1689 (52.0)	2264 (43.6)	
High school/GED	1558 (21.9)	485 (19.5)	1073 (23.1)	
Some college	1229 (21.7)	460 (19.8)	769 (22.7)	
College graduate or above	547 (9.8)	195 (8.4)	352 (10.6)	
*Health Insurance*				< .0001
Yes	4,311 (56.6)	1,876 (61.4)	2,435 (54.3)	
No	2,934 (43.4)	939 (38.6)	1,995 (45.7)	
**Poverty Income Ratio**	2.01 (0.04)	2.00 (0.04)	2.03 (0.05)	0.7132
**Current Smoker Status**	1233 (20.6)	498 (21.4)	735 (20.1)	< 0.0001
**Ever Cancer Diagnosis**	223 (2.41)	134 (4.2)	89 (1.5)	< 0.0001
**Ever Heart Attack**	162 (1.4)	113 (2.7)	49 (0.8)	< 0.0001
**Ever CHF**	131 (1.2)	101 (2.3)	30 (0.61)	< 0.0001
**High risk alcohol use**	1890 (26.0)	683 (9.3)	1207 (16.5)	0.0343

^*^Allostatic load (AL) was calculated as a cumulative index of biomarkers, with values ranging from 0 to 9. Scores of 0–2 were categorized as Low AL, and scores of 3 or higher were categorized as High AL

p-values determined from weighted Rao-Scott Chi-Square or weighted F-tests

### Outcome: cancer death

Our outcome of interest was cancer-related death. The NCHS provides public available linkages to the NDI through December 31, 2019. ICD-10 codes C00-C97 were concatenated by NDI for publicly available access and identified as deaths attributed to cancer. The NDI database contains death record information on file in state vital statistics offices [23]. Vital status death records were linked by NCHS using the NDI. NHANES collected either full nine-digit SSN or the last four digits. The NDI is a database of US death record information from state vital statistic offices, which includes underlying and multiple causes of death as well as identifying information to link records. This identifying information includes SSN, first name, middle initial, last name, father’s surname, birth month, birthday, birth year, sex, race, state or country of birth, and state of residence. Survey participant’s record was screened to match with the NDI based on at least one of the following combinations of identifying data elements 1) SSN, last name, first name 2) SSN, sex, birth month, birthday, birth year; or 3) last name, first name, birth month, and birth year.

### Sociodemographic characteristics and health behaviors

We examined sociodemographic characteristics and health behaviors of participants. We included age, sex, education, time-period surveyed, poverty to income ratio (PIR), citizenship status, length of time in the US, and country of birth. Education was categorized as less than high school, high school or GED, some college, or college graduate. The time period surveyed was divided into three intervals: 1999 – 2002, 2003 – 2006, and 2007 – 2010. Citizenship status was classified as US citizen, or non-US citizen based on the question “Are you a citizen of the United States?” Further, interviewers were cognizant and careful with this question regarding the sensitivity and fear of immigration retaliation and followed with “Providing this information is voluntary and is collected under the authority of the Public Health Service Act. There will be no effect on pending immigration or citizenship petitions.” Length of time in the US was categorized as less than 10 years, or greater than 10 years. Country of birth was recorded as the US, Mexico, or other based on NHANES questionnaire.

### Lifestyle factors and pre-existing conditions

In our analysis we examined possible health behaviors and comorbidities that may influence AL score including heart attack, congestive heart failure, smoker status, and alcohol use. We used NHANES questions that aligned with the AUDIT-C questionnaire to assess participants' risk of alcohol use (“How often did you have a drink containing alcohol in the past year?”, “On days in the past year when you drank alcohol how many drinks did you typically drink?”, and “How often did you have 5 or more drinks on an occasion in the past year?”). Women with an AUDIT-C score of 3 or higher and men with a score of 4 or higher were classified as high risk. Participants who had smoked fewer than 100 cigarettes in their lifetime were classified as never smokers. Those who had smoked 100 or more cigarettes but were not currently smoking were classified as past smokers. Participants who had smoked at least 100 cigarettes in their lifetime and were currently smoking were classified as current smokers.

### Statistical analysis

We conducted descriptive statistical analysis, calculating relative frequencies and presenting them as weighted percentages for categorical variables, and reporting means with associated standard deviations for continuous variables. NHANES applied sampling methods to adjust for unequal probabilities of selection, correct for nonresponse, and post stratify the weights to align with Census Bureau estimates of the US population. These adjustments were implemented at each survey stage: the screener, personal interview, and examination. To reduce respondent burden and streamline examinations, NHANES conducts subsampling for certain laboratory exams, measuring biomarkers in a random subset of participants. Subsample weights are then generated to account for selection probability and adjust for non-response bias.

The proportional hazards assumption was assessed for the primary exposure AL using a resampling-based supremum test of cumulative martingale residuals with 10,000 simulations. No statistically significant violations of the proportional hazards assumption were observed (all p-values > 0.05). We used Rao-Scott Chi-square tests to compare the descriptive statistics by AL status for categorical variables, and weighted Wald F-tests for continuous variables. Effect measure modification was assessed on the multiplicative scale by including cross-product interaction terms between AL and nativity-related factors (US citizenship status and length of time in the US) in survey-weighted Cox proportional hazards models. Statistical significance of interaction was evaluated using Wald tests (p-value < 0.10 for multiplicative interaction). Kaplan–Meier survival estimator was used to examine the survival function of cancer mortality by AL status overall and stratified by US citizenship status, length of time in the US, and country of birth (secondary analysis, results presented in Supplemental Tables 2–5).

Survival analyses were conducted using weighted Cox Proportional Hazard regression models to estimate hazard ratios (HRs) and 95% confidence intervals (CIs) for cancer mortality. Given the strong association between age and cancer mortality, we implemented a sequential modeling strategy. Model 1 was specified as an unadjusted model, estimating crude association between AL and cancer mortality. Model 2 was an age-adjusted model, with age included as a continuous variable (per 10-year increase) to account for confounding by age. Model 3 was a fully adjusted model, additionally accounting for sociodemographic factors (sex, education, country of birth, age, education level, and survey period) and health-related factors (smoking status, history of heart attack, and congestive heart failure).

We evaluated effect modification by nativity-related factors by testing interactions between AL and (1) US citizenship status (2) length of time in US, and (3) country of birth. To further examine heterogeneity by age, we conducted stratified analyses among individuals aged 40–59, and ≥ 60 years. Participants younger than 40 years were excluded from age-stratified analyses due to the relatively low incidence of cancer mortality; the average age in this group was 27 years, limiting the relevance and interpretability of long-term cancer mortality outcomes. This approach allowed us to explore how nativity-related factors and AL may interact differently across the life course. To address potential bias due to pre-existing or undiagnosed cancer at baseline, we conducted sensitivity analysis excluding participants with less than two years of follow-up from baseline. All analysis was conducted using SAS version 9.4 (Cary, North Carolina, USA) and Stata version 18.0 (College Station, Texas, USA).

## Results

**Fig. 1:**
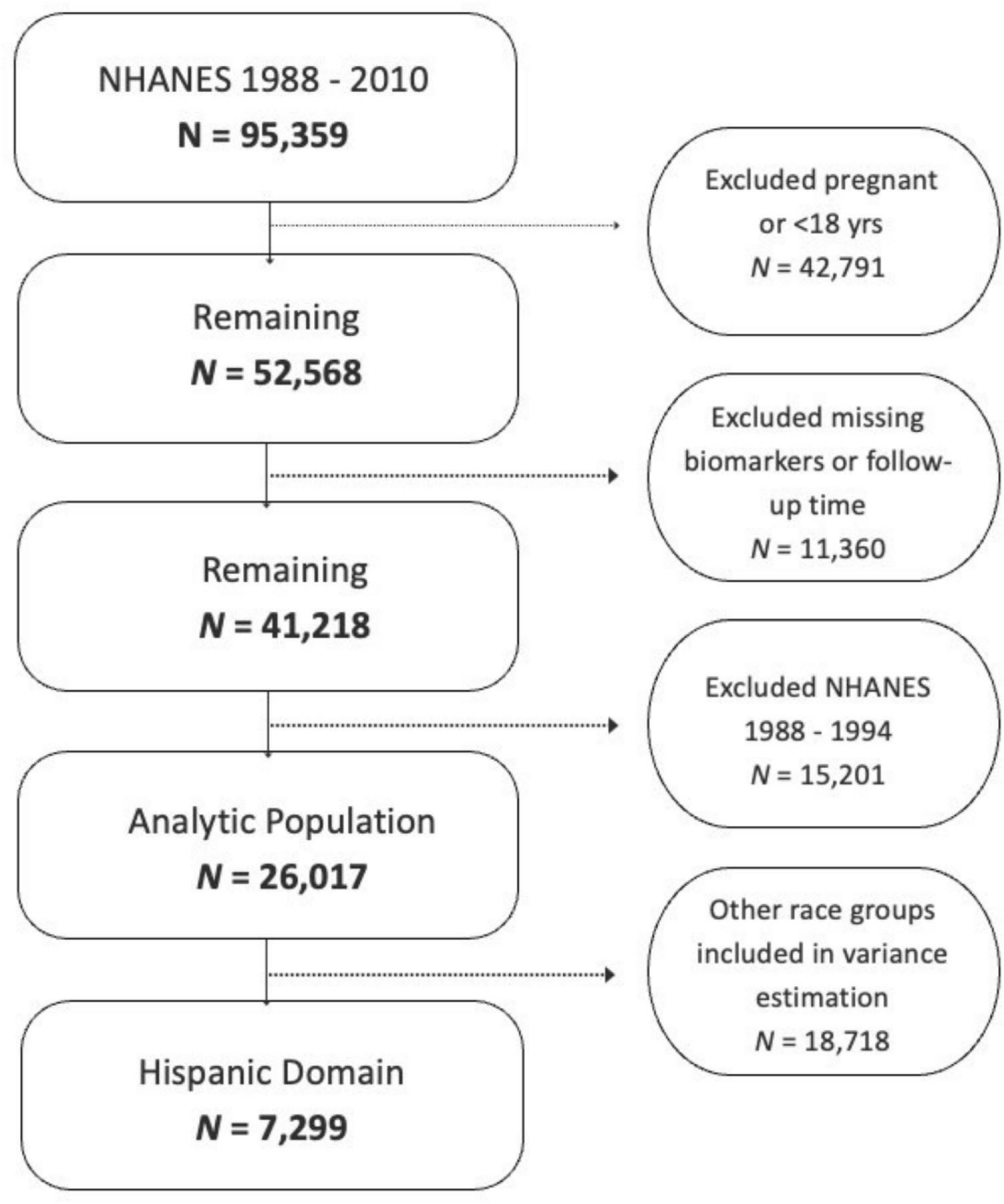
Flowchart of exclusion criteria

Table 1 presents the demographic characteristics of 7,299 Hispanic NHANES participants weighted sample corresponding to 22,420,430 people (See Fig. 1). The mean total allostatic score was 1.94 (SD = 0.04). Among the sample, 2,835 participants (33.4% weighted) had high AL (score ≥ 3), while 4,464 (66.6% weighted) had low AL (score < 3). Individuals with high AL were more likely to be older (mean age: 47.3 years vs 35.2 years) and have less than a high school education (52.0% vs. 43.6%, p-value < 0.001). They were also more likely to hold US citizenship (65% vs. 54.7%, p-value < 0.001) and to have lived in the US for more than 10 years (45% vs. 34.3%, p-value < 0.001). Regarding health behaviors and comorbidities, participants with a high AL were more likely to be current smokers (21.4% vs. 20.1%, p-value < 0.001), ever have cancer diagnosis (4.2% vs. 1.5%, p value < 0.001), more likely to ever had a heart attack (2.7% vs. 0.8%) and congestive heart failure (2.3% vs. 0.61%) compared to those with low AL.

**Fig. 2:**
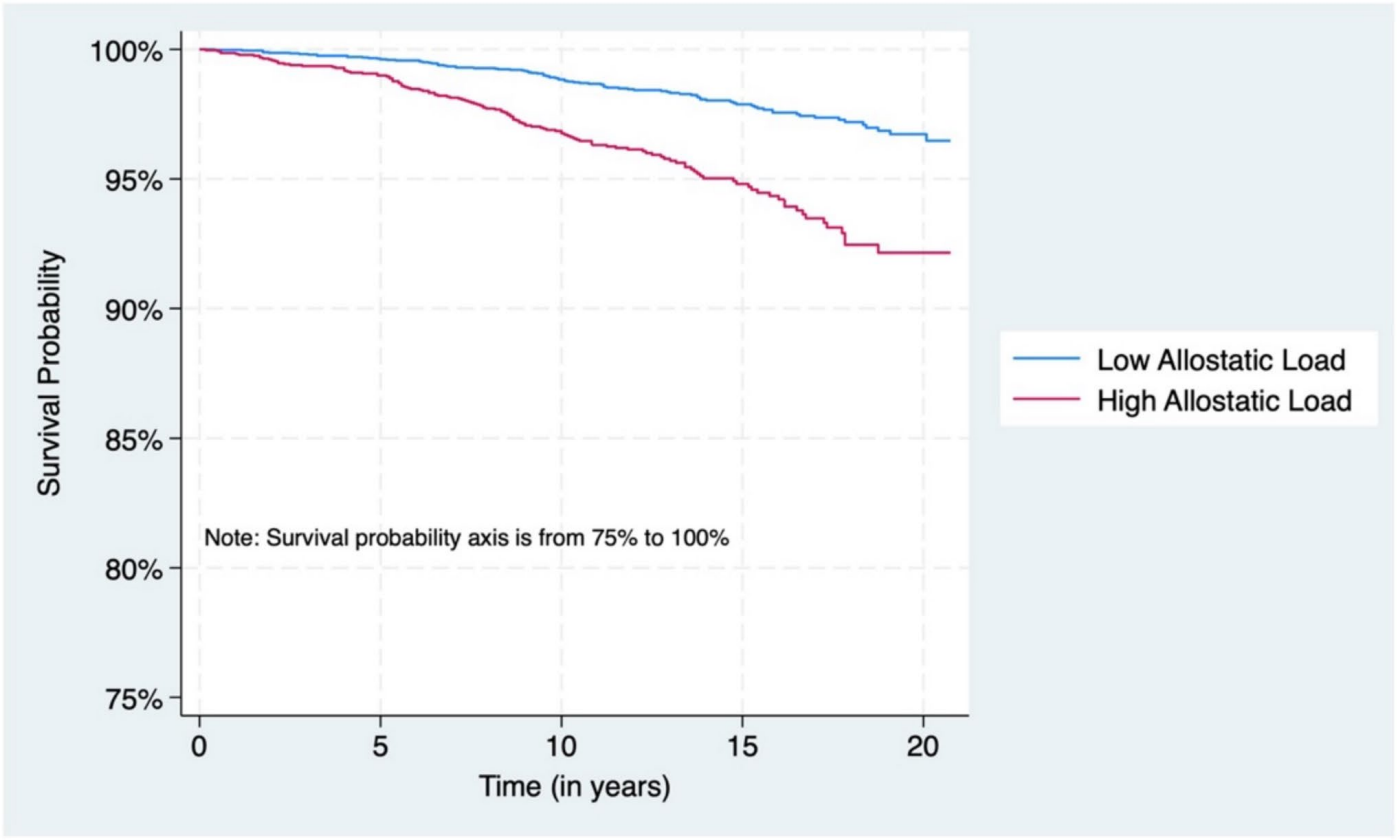
Kaplan–Meier survival plot of Cancer mortality by allostatic load status among Hispanic NHANES participants years 1999–2010

Across the full sample of 7,299 participants, the unadjusted model showed an increased risk of cancer mortality among individuals with high AL (Model 1 HR = 2.27, 95% CI = 1.51–3.40). (See Fig. 2 and Table 2). However, this association was attenuated and no longer statistically significant in fully adjusted models after accounting for age and in age-stratified analyses. Among individuals aged 60 years and older, adjusted models revealed statistically significant associations between US citizenship status and cancer mortality risk, regardless of AL status. Both, US citizens with high AL (Model 2 HR = 3.32, 95% CI = 1.18–9.35) and low AL (Model 2 HR = 3.13, 95% CI = 1.37–7.12) had higher cancer-related mortality compared to the reference group, non-US citizens with low AL (Table 3). Similarly, among adults aged ≥ 60 years, those who had lived in the US for more than 10 years exhibited a significantly increased risk of cancer mortality regardless of AL status. These findings were not apparent among individuals aged 40–59 years (Table 4).

**Table 2:** Weighted Cox Proportional Hazard Models with corresponding Hazard Ratios (HRs) and associated 95% Confidence Intervals (CIs) for the association between allostatic load (AL) and cancer-related mortality among Hispanic adults in NHANES 1999 – 2010 (follow-up through December 31, 2019), including full sample (N = 7,299) and age-stratified analyses

	No. of Cancer Deaths	Weighted No. of cancer deaths (%)	Model 1: Unadjusted HR (95% CI)	Model 2: Age-Adjusted HR (95% CI)	Model 3: Fully Adjusted HR (95% CI)
**Among full sample (N = 7,299)**					
Low AL	93	214,935 (49.7)	1.00 (Referent)	1.00 (Referent)	1.00 (Referent)
High AL	130	217,124 (50.3)	**2.27 (1.51–3.40)**	0.90 (0.59–1.37)	0.98 (0.62–1.55)
AL, per unit increase			**1.29 (1.18–1.42)**	0.97 (0.86–1.10)	0.98 (0.86–1.12)
**Among 40–59 years old (N = 2,163)**					
Low AL	25	82,019 (2.17)	1.00 (Referent)	1.00 (Referent)	1.00 (Referent)
High AL	27	65,387 (1.94)	0.96 (0.58–1.59)	0.75 (0.42–1.32)	0.90 (0.52–1.54)
AL, per unit increase			0.96 (0.80–1.15)	0.87 (0.70–1.09)	0.85(0.68–1.07)
**Among 60 + years old (N = 1,847)**					
Low AL	61	92,122 (10.29)	1.00 (Referent)	1.00 (Referent)	1.00 (Referent)
High AL	101	140,976 (8.61)	0.95 (0.51–1.77)	0.94 (0.50–1.77)	1.06 (0.56–2.03)
AL, per unit increase			0.97 (0.81–1.16)	0.97 (0.81–1.16)	1.01 (0.83–1.23)
**p-value for multiplicative interaction**			0.95	0.92	0.92

Model 1: Unadjusted/Crude

Model 2: Adjusted for age

Model 3: Model 2 + additional adjustment for sex, education, year interviewed, smoking status, history of heart attack, and congestive heart failure

Bolded hazard ratios indicate statistical significance

Multiplicative interaction tests for age-categories*allostatic load on model related outcome (cancer mortality)

**Table 3:** Weighted Cox Proportional Hazard Models with corresponding Hazard Ratios (HRs) and associated 95% Confidence Intervals (CIs) for the association between allostatic load with citizenship status on hazard of cancer-related mortality among Hispanic adults in NHANES 1999 – 2010 (follow-up through December 31, 2019), including full sample (N = 7,299) and age-stratified analyses

	No. of cancer deaths	Weighted No. of cancer deaths (%)	Model 1: Unadjusted HR (95% CI)	Model 2: Age-Adjusted HR (95% CI)	Model 3: Fully Adjusted HR (95% CI)
**Among full sample (N = 7,299)**					
Low AL & Non-US Citizen	29	79,800 (18.5)	1.00 (Referent)	1.00 (Referent)	1.00 (Referent)
Low AL & US Citizen	64	135,136 (31.3)	1.38 (0.77–2.46)	0.78 (0.42–1.47)	0.92 (0.52–1.63)
High AL & Non-US Citizen	26	46,186 (1.8)	1.66 (0.97–2.84)	0.60 (0.33–1.09)	2.68 (0.93–7.72)
High AL & US Citizen	102	169,318 (3.5)	**3.28 (1.91–5.64)**	0.81 (0.44–1.48)	0.98 (0.52–1.86)
**40–59 years old (N = 2,163)**					
Low AL & Non-US Citizen	14	49,400 (3.2)	1.00 (Referent)	1.00 (Referent)	1.00 (Referent)
Low AL & US Citizen	11	32,619 (1.5)	**0.41 (0.17–0.95)**	**0.33 (0.14–0.79)**	0.50 (0.21–1.14)
High AL & Non-US Citizen	11	26,021 (2.1)	0.73 (0.34–1.57)	0.56 (0.25–1.27)	0.72 (0.33–1.56)
High AL & US Citizen	16	39,366 (1.8)	0.55 (0.28–1.09)	**0.35 (0.16–0.79)**	0.61 (0.31–1.21)
**60+ years old (N =1,847)**					
Low AL & Non-US Citizen	9	7,605 (4.2)	1.00 (Referent)	1.00 (Referent)	1.00 (Referent)
Low AL & US Citizen	52	84,517 (11.8)	**3.06 (1.32–7.09)**	**2.92 (1.24–6.86)**	**3.13 (1.37–7.12)**
High AL & Non-US Citizen	14	13,156 (3.5)	0.92 (0.33–2.52)	0.92 (0.34–2.52)	1.06 (0.36–3.12)
High AL & US Citizen	85	126,200 (10.1)	**2.98 (1.18–7.51)**	**2.80 (1.10–7.16)**	**3.32 (1.18–9.35)**
**p-value for multiplicative interaction**			< .0001	< .0001	< .0001

Model 1: Unadjusted/Crude

Model 2: Adjusted for age

Model 3: Model 2 + additional adjustments for sex, education, year interviewed, smoking status, history of heart attack, and congestive heart failure

Bolded hazard ratios indicate statistical significance

Multiplicative interaction tests for age-categories*allostatic load* US citizenship on model related outcome (cancer mortality)

**Table 4:** Weighted Cox Proportional Hazard Models with corresponding Hazard Ratios (HRs) and associated 95% Confidence Intervals (CIs) for the association between allostatic load with length of time in the US on hazard of cancer-related mortality among Hispanic adults in NHANES 1999 – 2010 (follow-up through December 31, 2019), including full sample (N = 7,299) and age-stratified analyses

	No. of Cancer Deaths	Weighted No. of cancer deaths (%)	Model 1: Unadjusted HR (95% CI)	Model 2: Age-Adjusted HR (95% CI)	Model 3: Fully Adjusted HR (95% CI)
**Among full sample (N =7,299)**					
Low AL and < 10 years	11	30,553 (0.8)	1.00 (Referent)	1.00 (Referent)	1.00 (Referent)
High AL and < 10 years	6	17,817 (1.9)	**2.57 (1.14–5.77)**	0.83 (0.36–1.93)	1.03 (0.45–2.40)
Low AL and $\ge$ 10 years	43	105,030 (2.1)	**2.72 (1.21–6.12)**	0.99 (0.43–2.32)	1.09 (0.45–2.61)
High AL and $\ge$ 10 years	60	110,805 (3.3)	**4.91 (2.33–10.35)**	0.89 (0.40–1.96)	1.04 (0.44–2.44)
**Among 40–59 years old (N = 2,163)**					
Low AL and < 10 years	5	15,132 (2.7)	1.00 (Referent)	1.00 (Referent)	1.00 (Referent)
High AL and < 10 years	2	5,000 (1.6)	0.60 (0.11–3.27)	0.34 (0.06–1.90)	0.58 (0.10–3.33)
Low AL and $\ge$ 10 years	12	42,537 (2.2)	0.76 (0.23–2.53)	0.50 (0.17–1.49)	0.78 (0.25–2.40)
High AL and $\ge$ 10 years	16	36,114 (2.0)	0.74 (0.29–1.89)	0.41 (0.15–1.10)	0.71 (0.27–1.87)
**Among 60 + years old (N = 1,847)**					
Low AL and < 10 years	2	1,106 (1.7)	1.00 (Referent)	1.00 (Referent)	1.00 (Referent)
High AL and < 10 years	3	5,808 (5.2)	3.81 (0.59–24.66)	3.64 (0.57–23.48)	4.20 (0.69–25.51)
Low AL and $\ge$ 10 years	29	54,012 (10.9)	**7.91 (1.70–36.86)**	**7.87 (1.62–38.23)**	**6.32 (1.33–29.9)**
High AL and $\ge$ 10 years	44	74,691 (8.4)	**6.92 (1.30–36.92)**	**6.95 (1.30–36.56)**	**6.34 (1.17–34.43)**
**p-value for multiplicative interaction**			**< .0001**	**< .0001**	** < .0001**

Model 1: Unadjusted/Crude

Model 2: Adjusted for age

Model 3: Model 2 + additional adjustments for sex, education, year interviewed, smoking status, history of heart attack, and congestive heart failure

Bolded hazard ratios indicate statistical significance

Multiplicative interaction tests for age-categories*allostatic load* length of time in US on model related outcome (cancer mortality)

### Secondary and sensitivity analyses

We provide findings for interaction between AL and country of birth and their effects on cancer mortality in Supplemental Table 2. In unadjusted analysis, participants with high AL, regardless of country of birth, had twofold increased risks for cancer mortality as compared to participants with low AL. These effects attenuated after adjustments for potential confounders. Supplemental Tables 3–5 provide results excluding participants with less than 2 years of follow-up to account for those with underlying cancer (or other severe illnesses). These findings mirrored the results of primary analysis, with most significant findings occurring among our age-stratified analysis (those aged 60 +), and interaction between AL and length of time in US; participants living in the US greater than 10 years had sixfold higher risk for cancer mortality whether they had low AL (Supplemental Table 5, Model 3 HR = 6.44, 95% CI = 1.32 – 31.54), or high AL (Model 3 HR = 6.55, 95% CI = 1.17 – 36.68) as compared to participants with low AL and less than 10 years living in the US.

## Discussion

This study investigated the relationship between AL and cancer mortality among Hispanic adults, with a focus on US citizenship status and length of time in the US. Our findings indicate that cancer mortality risk was most pronounced among older adults (≥ 60 years), particularly among those who were US citizens or had lived in the US for more than 10 years. Importantly, these associations were observed regardless of AL status, suggesting the possibility that nativity-related factors and cumulative exposure to US social and structural contexts may play a role in cancer mortality risk among older Hispanic adults other than AL alone. Although elevated AL was associated with increased cancer mortality in unadjusted analyses among Hispanics 18 years and older, this relationship was largely attenuated after accounting for age and other confounders.

One possible explanation of this is the Salmon Bias Hypothesis which suggests immigrants who become seriously ill or approach the end of life may return to their country of origin [6]. This phenomenon is not specific to individuals with higher AL, but may differentially affect observed mortality patterns by nativity and duration of US residence, particularly among older adults. As a result, cancer deaths among foreign-born individuals with shorter US residence may be undercaptured, while mortality risk appears elevated among those with prolonged residence or US citizenship, particularly at older ages. This pattern was age-specific. Among individuals aged 40–59 years, US citizenship was associated with lower cancer mortality risk across both low and high AL groups, suggesting a protective effect of citizenship and/or healthcare access earlier in the life course. In contrast, among individuals aged 60 years and older, US citizenship and longer US residence were associated with substantially higher cancer mortality risk, regardless of AL status.

These contrasting age-specific associations suggest that the health implications of US citizenship and prolonged residence may differ across the life course. Protective associations observed in midlife may reflect improved access to preventive care, screening, and earlier-stage diagnosis among citizens. However, among older adults, cumulative exposure to structural stressors, acculturative processes, and selective return migration consistent with the Salmon Bias Hypothesis may contribute to an apparent concentration of cancer mortality among long-term residents and US citizens. Individuals younger than 40 years did not exhibit elevated cancer mortality risk, consistent with the low incidence of cancer-related deaths in this age group and limited power to detect differences. Previous findings, like those from the Texas City Stress and Health Study, indicate that US-born Mexican have higher AL scores than foreign-born Mexicans, who are also least likely to fall into higher AL categories [24]. Findings from the Hispanic Community Healthy Study/Study of Hispanic reported among individuals under 55, AL was highest in US-born Hispanic individuals, intermediate in foreign-born Hispanic with 10 or more years in the US, and lowest in those with less than 10 years in the US, with AL also increasing among those who immigrated at a younger age; trends were less pronounced in those 55 and older [25]. These studies underscore how negative acculturation in the US influences cancer mortality outcomes. These results partly align with the “Healthy Immigrant Effect,” which posits that immigrants initially have better health profiles that erode over time, potentially due to acculturative stress, adoption of unhealthy behaviors, or systemic barriers to care [26].

While citizenship, whether by birth or naturalization, might be expected to improve access to healthcare and resources that reduce stress levels and mitigate cancer risks, many Hispanic individuals still do not fully experience these benefits. Potochnick et al. (2024) describes that restrictive immigration policies create a “chilling effect” on healthcare access, discouraging even US-born Hispanic people with undocumented family members from seeking essential health services, including preventive care [27]. Additionally, growing deportation fears among Hispanic US citizens, particularly since the 2016 US presidential election, reflect a heightened awareness of immigration policies and perceived hostility toward immigrant communities [28]. Together, these factors contribute to an avoidance of healthcare services, leading to missed preventative measures, such as vaccinations, screenings, and early cancer interventions, contributing to elevated cancer mortality risk in these populations [29]. When Hispanic individuals with Medicare do seek care, they report poorer experiences with accessing needed services, timely care and care coordination compared to non-Hispanic White counterparts, with additional disparities in doctor communication and customer service, particularly in rural areas [30].

For individuals who obtain citizenship status by naturalization, prolonged time spent in the US may contribute to increased AL due to acculturative stress and the adoption of unhealthy behaviors. Yellow Horse et al. (2019) suggests that foreign-born Hispanic adults who have lived in the US for 20 or more years have AL scores similar to those US-born individuals [31].

As reported by the American Cancer Society, Hispanic individuals diagnosed with cancer often face significant financial challenges that may impact treatment access and survivorship. Approximately, 28% of Hispanic individuals were uninsured, compared with 8% in non-Hispanic White individuals [32]. This disparity in insurance coverage contributes to disproportionately higher medical costs for Hispanic patients, which exacerbates existing health disparities and limits access to adequate care. These financial burdens are not just isolated to treatment costs but also extend to long-term survivorship, where ongoing care and monitoring are crucial. Hispanic individuals are frequently studied as a homogenous group in epidemiologic and clinical research; however, this population is remarkably diverse, encompassing various nativity statuses, countries of origin, races, religions, languages, and cultures [13]. For example, established groups such as Cubans and Puerto Ricans exhibit notably higher rates of obesity-related cancer mortality, including colorectal and endometrial cancers [33]. In contrast, newer immigrant groups present cancer patterns akin to those found in their countries of origin, with elevated rates of infection-related cancers such as stomach, liver, cervical, and gallbladder cancers [34].

## Strengths and limitations

This study presents several strengths, but it is also essential to acknowledge its limitations. A key strength is the use of NHANES, which provides a nationally representative sample that allows us to examine the relationship between AL and cancer mortality across diverse US adult populations. The NHANES survey also oversamples adults 60 and older, as well as Hispanic participants, enhancing our ability to generalize findings across these groups. Furthermore, 20 years of follow-up data strengthen our analysis, providing a robust timeframe for mortality outcomes.

However, NHANES is a cross-sectional survey and was not originally designed to track cancer incidence or outcomes. Consequently, we lacked detailed data on cancer type, incidence, treatment, and progression, prevent us from assessing cancer incidence, survival, and potential differences in detection, screening, or treatment. Additionally, NHANES used standardized methods for biomarker collection and gathered data on various health behaviors and conditions, which likely minimized misclassification of our primary exposure. However, the potential for measurement error in these variables remains. Additionally, several stratified analyses were based on relatively small sample sizes and limited numbers of cancer-related deaths, which may have contributed to unstable estimates and wider confidence intervals.

Age remains a critical confounder in analyzing the relationship between immigration-related factors, AL, and cancer mortality risk. Although we stratified by age groups to account for life-course differences, younger individuals may not have lived long enough to develop or die from cancer, potentially underestimating associations in these groups. Additionally, AL was only measured once at baseline, which may not reflect cumulative physiological stress over time or changes due to evolving lifestyle, environmental exposures, and coping mechanisms. In this analysis of publicly available NHANES-NDI linked data, we did not assess risks for site specific cancers. Future research should focus on older populations and leverage longitudinal data to more accurately assess the long-term effects of AL on cancer outcomes and specifically elucidate risks by cancer types. NHANES does not allow for detailed disaggregation of Hispanic populations (e.g., limited to Mexican American or Other Hispanic), which precludes examination of differences across diverse Hispanic subgroups. Despite these limitations, our study contributes to an understudied area, highlighting how immigration-related factors and AL intersect to influence cancer mortality risk among Hispanic populations—an interaction crucial to understanding long-term adaptation to the US environment.

## Conclusion

In this nationally representative cohort of Hispanic adults, we found that cancer mortality risk was strongly driven by age and certain nativity-related factors, rather than AL alone. In fully adjusted models, older age (≥ 60 years) was consistently associated with substantially higher cancer mortality risk, particularly among individuals who were US citizens or who had lived in the US for more than 10 years. These associations persisted regardless of AL status, suggesting that prolonged exposure to US social and structural contexts may play a critical role in shaping cancer mortality risk among older Hispanic adults.

## Supplementary Information

Below is the link to the electronic supplementary material.

Supplementary file1 (DOCX 36 KB)

## Data Availability

Data are publicly available and are accessed from NHANES website ([https://www.cdc.gov/nchs/nhanes/about_nhanes.htm]. (https:/www.cdc.gov/nchs/nhanes/about_nhanes.htm)).
