# Supplementary material for: Understanding the role of age and U.S. acculturation factors on the relationship between allostatic load and cancer mortality risk in Hispanic Americans
Source: Cancer Causes Control. 2026 Apr 22;37(6):86. doi: 10.1007/s10552-026-02162-z (PMC13102732; doi:10.1007/s10552-026-02162-z)
Supplement: Supplementary file 1 — Supplementary file1 (DOCX 36 KB) [file 10552_2026_2162_MOESM1_ESM.docx]

| **Supplemental Table 1:** NHANES 1999 – 2010 allostatic load biomarkers distribution and cut points for high/low classification. | | |
| --- | --- | --- |
| **Allostatic Load Biomarkers** | **Median (Q1, Q3)** | **High-Risk cutoff** |
| **Body Mass Index (kg/m2)** |  |  |
| Female | 26.19 (22.60, 31.24) | >31.24 |
| Male | 26.89 (24.00, 30.33) | >30.33 |
| **Systolic Blood Pressure (mmHg)** | | |
| Female | 116.70 (106.76, 131.41) | >131.41 |
| Male | 121.54 (112.96, 132.15) | >132.15 |
| **Diastolic Blood Pressure (mmHg)** | | |
| Female | 69.92 (62.82, 77.70) | >77.70 |
| Male | 73.27 (65.93, 81.07) | >81.07 |
| **C-reactive Protein (mg/dL)** | | |
| Female | 0.21 (0.13, 0.49) | >0.49 |
| Male | 0.20 (0.09, 0.30) | >0.30 |
| **Creatinine (mol/L)** |  |  |
| Female | 70.71 (61.49, 79.60) | >79.60 |
| Male | 88.21 (79.16, 103.21) | >103.21 |
| **Total Cholesterol (mg/dL)** | | |
| Female | 198.20 (172.58, 227.70) | >227.70 |
| Male | 196.28 (169.48, 225.11) | >225.11 |
| **Triglycerides, serum (mg/dL)** | | |
| Female | 103.63 (71.70, 154.97) | >154.97 |
| Male | 124.00 (83.03, 192.02) | >192.02 |
| **Albumin, serum (g/dL)** | | |
| Female | 4.14 (3.92, 4.35) | <4.35 |
| Male | 4.33 (4.12, 4.55) | <4.35 |
| **Glycohemoglobin (%)** | | |
| Female | 5.23 (4.96, 5.55) | > 5.55 |
| Male | 5.28 (5.01, 5.56) | > 5.56 |
| We defined high AL as having more than three abnormal measures among the following nine biomarkers: BMI, C-reactive protein (CRP), systolic blood pressure (SBP), diastolic blood pressure (DBP), glycated hemoglobin, total cholesterol, serum triglycerides, serum creatinine, and serum albumin. Sex-specific distributions were used to define high-risk thresholds for allostatic load biomarkers based on the full NHANES 1999–2010 sample. For BMI, CRP, systolic blood pressure, diastolic blood pressure, glycated hemoglobin, total cholesterol, serum triglycerides, and serum creatinine, values above the sex-specific 75th percentile (Q3) were classified as high risk. For serum albumin, values below the sex-specific 25th percentile (Q1) were classified as high risk. Median values and interquartile ranges (Q1, Q3) are shown for reference. | | |

| **Supplemental Table 2.** Weighted Cox Proportional Hazard Models with corresponding Hazard Ratios (HRs) and associated 95% Confidence Intervals (CIs) for the association between allostatic load with country of birth on hazard of cancer-related mortality among Hispanic adults in NHANES 1999 – 2010 (follow-up through December 31, 2019), including full sample (N = 7,299) and age-stratified analyses. | | | | | |
| --- | --- | --- | --- | --- | --- |
|  | **No. of Cancer Deaths** | **Weighted No. of cancer deaths** **(%)** | **Model 1:**  **Unadjusted**  **HR (95% CI)** | **Model 2:**  **Age-Adjusted**  **HR (95% CI)** | **Model 3:**  **Fully Adjusted**  **HR (95% CI)** |
| **Among full sample** | | | | | |
| Low AL and Mexico born | 41 | 69,822 (1.3) | 1.00 (Referent) | 1.00 (Referent) | 1.00 (Referent) |
| Low AL and US born | 37 | 76,739 (1.4) | 1.07 (0.57–2.03) | 0.97 (0.50–1.90) | 1.07 (0.61–1.92) |
| Low AL and Other born | 15 | 68,374 (1.7) | 1.30 (0.60–2.83) | 0.79 (0.36–1.74) | 0.86 (0.38–1.92) |
| High AL and Mexico born | 44 | 57,529 (2.3) | **2.02 (1.32–3.84)** | 0.80 (0.52–1.22) | 0.87 (0.57–1.33) |
| High AL and US born | 62 | 86,883 (2.9) | **2.56 (1.71–3.83)** | 0.90 (0.61–1.33) | 1.04 (0.67–1.64) |
| High AL and Other born | 23 | 71,521 (3.5) | **2.97 (1.55–5.71)** | 0.74 (0.41–1.34) | 0.90 (0.47–1.71) |
| **Among 40-59 years old (N = 2,163)** | | | | | |
| Low AL and Mexico born | 13 | 30, 976 (2.1) | 1.00 (Referent) | 1.00 (Referent) | 1.00 (Referent) |
| Low AL and US born | 8 | 24,350 (2.1) | 0.87 (0.40–1.91) | 0.73 (0.33–1.60) | 1.27 (0.57–2.81) |
| Low AL and Other born | 4 | 26,694 (2.3) | 0.94 (0.28–3.14) | 0.81 (0.25–2.68) | 1.07 (0.34–3.40) |
| High AL and Mexico born | 12 | 28,803 (2.4) | 1.20 (0.60–2.38) | 0.93 (0.46–1.90) | 1.21 (0.59–2.47) |
| High AL and US born | 9 | 24,273 (2.1) | 0.97 (0.43–2.20) | 0.63 (0.27–1.47) | 1.30 (0.52–3.23) |
| High AL and Other born | 6 | 12,311 (1.2) | 0.53 (0.23–1.20) | **0.36 (0.15–0.87)** | 0.62 (0.27–1.40) |
| **Among 60+ years old (N = 1,847)** | | | | | |
| Low AL and Mexico born | 22 | 22 (7.2) | 1.00 (Referent) | 1.00 (Referent) | 1.00 (Referent) |
| Low AL and US born | 28 | 28 (11.4) | 1.66 (0.70–3.89) | 1.55 (0.64–3.71) | 1.59 (0.72–3.51) |
| Low AL and Other born | 11 | 11 (11.2) | 1.40 (0.53–3.70) | 1.42 (0.52–3.83) | 1.30 (0.55–3.07) |
| High AL and Mexico born | 32 | 32 (6.6) | 1.00 (0.52–1.92) | 1.03 (0.53–2.00) | 1.07 (0.54–2.13) |
| High AL and US born | 52 | 52 (9.9) | 1.58 (0.82–3.04) | 1.50 (0.77–2.93) | 1.61 (0.80–3.22) |
| High AL and Other born | 16 | 16 (8.7) | 1.27 (0.51–3.20) | 1.22 (0.48–3.10) | 1.40 (0.51–3.88) |
| **p-value for multiplicative interaction** | | | <.0001 | <.0001 | <.0001 |
| Model 1: Unadjusted/Crude.  Model 2: Adjusted for age (except in models stratified by age group).  Model 3: Model 2 + additional adjustment for sex, education, year interviewed, smoking status, history of heart attack, and congestive heart failure.  **Bolded hazard ratios indicate statistical significance.**  Multiplicative interaction tests for age-categories*allostatic load*country of birth on model related outcome (cancer mortality) | | | | | |

| **Supplemental Table 3.** Weighted Cox Proportional Hazard Models with corresponding Hazard Ratios (HRs) and associated 95% Confidence Intervals (CIs) for the association between allostatic load and cancer-related mortality among Hispanic adults in NHANES 1999 – 2010 (follow-up through December 31, 2019), including full sample (N = 7,227) and age-stratified analyses. ^1^Among participants with 2 years or more of follow up. | | | | | |
| --- | --- | --- | --- | --- | --- |
|  | **No. of Cancer Deaths** | **Weighted No. of cancer deaths (%)** | **Model 1:**  **Unadjusted**  **HR (95% CI)** | **Model 2: Age-Adjusted HR (95% CI)** | **Model 3:**  **Fully Adjusted**  **HR (95% CI)** |
| **Among full sample (N = 7,227)** | | | | | |
| Low Allostatic Load | 87 | 207,953 (50.6) | 1.00 (Referent) | 1.00 (Referent) | 1.00 (Referent) |
| High Allostatic Load | 119 | 203,007 (49.4) | **2.20 (1.46–3.40)** | 0.88 (0.57–1.37) | 0.96 (0.59–1.54) |
| AL, per unit increase |  | | **1.29 (1.18–1.42)** | 0.98 (0.86–1.12) | 0.99 (0.86–1.13) |
| **40-59 years old (N = 2,152)** | | | | | |
| Low Allostatic Load | 23 | 77,907 (55.1) | 1.00 (Referent) | 1.00 (Referent) | 1.00 (Referent) |
| High Allostatic Load | 26 | 63,504 (44.9) | 0.99 (0.58–1.67) | 0.77 (0.43–1.38) | 0.91 (0.52–1.59) |
| AL, per unit increase |  | | 0.98 (0.81–1.17) | 0.89 (0.71–1.21) | 0.87 (0.69–1.10) |
| **60+ years old (N = 1,790)** | | | | | |
| Low Allostatic Load | 57 | 89,251 (40.9) | 1.00 (Referent) | 1.00 (Referent) | 1.00 (Referent) |
| High Allostatic Load | 91 | 128,743 (59.1) | 0.90 (0.46–1.75) | 0.89 (0.46–1.75) | 1.00 (0.50–2.02) |
| AL, per unit increase |  | | 0.97 (0.80–1.17) | 0.96 (0.79–1.17) | 1.00 (0.81–1.24) |
| **p-value for multiplicative interaction** | | | 0.93 | 0.98 | 0.94 |
| Model 1: Unadjusted/Crude.  Model 2: Adjusted for age (except in models stratified by age group).  Model 3: Model 2 + Additionally adjusted for sex, education, year interviewed, smoking status, history of heart attack, and congestive heart failure. **Bolded hazard ratios indicate statistical significance.**  Multiplicative interaction tests for age-categories*allostatic load on model related outcome (cancer mortality).  ^1^This analysis was restricted to NHANES participants with greater than 2 years of follow up to potentially account for cancer participants with underlying metastatic disease. | | | | | |

| **Supplemental Table 4.** Weighted Cox Proportional Hazard Models with corresponding Hazard Ratios (HRs) and associated 95% Confidence Intervals (CIs) for the association between allostatic load with citizenship status on hazard of cancer-related mortality among Hispanic adults in NHANES 1999 – 2010 (follow-up through December 31, 2019), including full sample (N = 7,227) and age-stratified analyses. ^1^Among participants with 2 years or more of follow up. | | | | | |
| --- | --- | --- | --- | --- | --- |
|  | **No. of Cancer Deaths** | **Weighted No. of cancer deaths**  **(%)** | **Model 1:**  **Unadjusted**  **HR (95% CI)** | **Model 2: Age-Adjusted HR (95% CI)** | **Model 3:**  **Fully Adjusted**  **HR (95% CI)** |
| **Among full sample (N = 7,227)** | | | | | |
| Low AL & Non-US Citizen | 28 | 78,058 (19.0) | 1.00 (Referent) | 1.00 (Referent) | 1.00 (Referent) |
| Low AL & US Citizen | 59 | 129,895 (31.6) | 1.36 (0.75–2.44) | 0.78 (0.41–1.48) | 0.91 (0.50–1.63) |
| High AL & Non-US Citizen | 25 | 45,834 (11.2) | 1.69 (0.99–2.89) | 0.62(0.35–1.13) | 0.75 (0.40–1.40) |
| High AL & US Citizen | 93 | 155,982 (38.0) | **3.11 (1.79–5.39)** | 0.78(0.42–1.45) | 0.94 (0.49–1.82) |
| **40-59 years old (N = 2,152)** | | | | | |
| Low AL & Non-US Citizen | 13 | 47,659 (33.7) | 1.00 (Referent) | 1.00 (Referent) | 1.00 (Referent) |
| Low AL & US Citizen | 10 | 30,248 (21.4) | **0.39 (0.16–0.94)** | **0.32 (0.13–0.78)** | 0.48 (0.20–1.16) |
| High AL & Non-US Citizen | 11 | 26,021 (18.4) | 0.76 (0.34–1.67) | 0.59 (0.25–1.36) | 0.74 (0.33–1.65) |
| High AL & US Citizen | 15 | 37,483 (26.5) | 0.54 (0.27–1.10) | **0.34 (0.15–0.78)** | 0.61 (0.30–1.24) |
| **60+ years old (N = 1,790)** | | | | | |
| Low AL & Non-US Citizen | 9 | 7,605 (3.5) | 1.00 (Referent) | 1.00 (Referent) | 1.00 (Referent) |
| Low AL & US Citizen | 48 | 81,646 (37.5) | **2.97 (1.27–6.95)** | **2.85 (1.20–6.77)** | **3.03 (1.31–7.00)** |
| High AL & Non-US Citizen | 13 | 12,804 (5.9) | 0.90 (0.32–2.52) | 0.90(0.32–2.51) | 1.04 (0.34–3.14) |
| High AL & US Citizen | 77 | 114,748 (52.6) | **2.74 (1.06–7.09)** | **2.60(1.00–6.81)** | **3.03 (1.03–8.93)** |
| **p-value for multiplicative interaction** | | | **<.0001** | **<.0001** | **<.0001** |
| Model 1: Unadjusted/Crude.  Model 2: Adjusted for age (except in models stratified by age group),  Model 3: Model 2 + Additionally adjusted for sex, education, year interviewed, smoking status, history of heart attack, and congestive heart failure.  **Bolded hazard ratios indicate statistical significance.**  ^1^This analysis was restricted to NHANES participants with greater than 2 years of follow up to potentially account for cancer participants with underlying metastatic disease.  Multiplicative interaction tests for age-categories*allostatic load*citizenship-status on model related outcome (cancer mortality). | | | | | |

| **Supplemental Table 5.** Weighted Cox Proportional Hazard Models with corresponding Hazard Ratios (HRs) and associated 95% Confidence Intervals (CIs) for the association between allostatic load with length of time in the US on hazard of cancer-related mortality by among Hispanic adults in NHANES 1999 – 2010 (follow-up through December 31, 2019), including full sample (N = 7,227) and age-stratified analyses. | | | | | |
| --- | --- | --- | --- | --- | --- |
|  | **No. of Cancer Deaths** | **Weighted No. of cancer deaths** **(%)** | **Model 1:**  **Unadjusted**  **HR (95% CI)** | **Model 2:**  **Age-Adjusted**  **HR (95% CI)** | **Model 3:**  **Fully Adjusted**  **HR (95% CI)** |
| **Among full sample (N = 7,227)** | | | | | |
| Low AL and Less than 10 years | 11 | 30,553 (7.4) | 1.00 (Referent) | 1.00 (Referent) | 1.00 (Referent) |
| High AL and Less than 10 years | 5 | 16,147 (3.9) | **2.34 (0.97–5.63)** | 0.78 (0.31–1.93) | 0.96 (0.39–2.39) |
| Low AL and Greater than 10 years | 40 | 102,299 (24.9) | **2.65 (1.16–6.07)** | 0.99 (0.42–2.35) | 1.09 (0.45–2.64) |
| High AL and Greater than 10 years | 58 | 110,007 (26.8) | **4.91 (2.34–10.33)** | 0.92 (0.44–1.94) | 1.07 (0.45–2.44) |
| **Among 40-59 years old (N = 2,163)** | | | | | |
| Low AL and Less than 10 years | 5 | 15,132 (10.7) | 1.00 (Referent) | 1.00 (Referent) | 1.00 (Referent) |
| High AL and Less than 10 years | 2 | 5,000 (3.5) | 0.60 (0.11–3.28) | 0.34 (0.06–1.91) | 0.58 (0.10–3.34) |
| Low AL and Greater than 10 years | 11 | 40,795 (28.8) | 0.72 (0.21–2.46) | 0.48 (0.16–1.44) | 0.75 (0.24–2.32) |
| High AL and Greater than 10 years | 16 | 36,114 (25.5) | 0.74 (0.29–1.90) | 0.41 (0.51–1.10) | 0.70 (0.27–1.85) |
| **Among 60+ years old (N = 1,847)** | | | | | |
| Low AL and Less than 10 years | 2 | 1,106 (0.5) | 1.00 (Referent) | 1.00 (Referent) | 1.00 (Referent) |
| High AL and Less than 10 years | 2 | 4,138 (1.9) | 2.75 (0.35–21.64) | 2.65 (0.34–20.79) | 3.10 (0.42–22.96) |
| Low AL and Greater than 10 years | 27 | 53,023 (24.3) | **7.86 (1.67–37.02)** | **7.86 (1.61–38.47)** | **6.44 (1.32–31.54)** |
| High AL and Greater than 10 years | 42 | 73893 (33.9) | **6.99 (1.30–37.61)** | **6.96 (1.31–36.96)** | **6.55 (1.17–36.68)** |
| **p-value for multiplicative Interaction** | | | **<.0001** | **<.0001** | **<.0001** |
| Model 1: Unadjusted/Crude.  Model 2: Adjusted for age (except in models stratified by age group).  Model 3: Model 2 + additionally adjusted for sex, education, year interviewed, smoking status, history of heart attack, and congestive heart failure.  **Bolded hazard ratios indicate statistical significance.**  ^1^This analysis was restricted to NHANES participants with greater than 2 years of follow up to potentially account for cancer participants with underlying metastatic disease. Multiplicative interaction tests for age-categories*allostatic load*length of time in US on model related outcome (cancer mortality). | | | | | |
